# Drug-induced hepatitis superimposed on the presence of anti-SLA antibody: a case report

**DOI:** 10.1186/1752-1947-2-25

**Published:** 2008-01-28

**Authors:** Aitziber Etxagibel, M Rosa Julià, Alvaro Brotons, M Margarita Company, Carlos Dolz

**Affiliations:** 1 Department of Family Practice Post-graduate Unit, Palma de Mallorca, Spain; 2 Immunology Service, Son Dureta Hospital, Palma de Mallorca, Spain; 3 Gastroenterology and Hepatology Department, Son Llàtzer Hospital, Palma de Mallorca, Spain; 4 Pathology Department, Son Llàtzer Hospital, Palma de Mallorca, Spain

## Abstract

**Introduction:**

Autoimmune hepatitis is a necroinflammatory disorder of unknown etiology characterized by the presence of circulating antibodies, hypergammaglobulinemia, and response to immunosuppression. It has the histological features of chronic hepatitis. The onset is usually insidious, but in some patients the presentation may be acute and occasionally severe. Certain drugs can induce chronic hepatitis mimicking autoimmune hepatitis. Different autoantibodies have been associated with this process but they are not detectable after drug withdrawal and clinical resolution.

**Case presentation:**

We describe a case of drug-induced acute hepatitis associated with antinuclear, antisoluble liver-pancreas and anti-smooth muscle autoantibodies in a 66-year-old woman. Abnormal clinical and biochemical parameters resolved after drug withdrawal, but six months later anti-soluble liver-pancreas antibodies remained positive and liver biopsy showed chronic hepatitis and septal fibrosis. Furthermore, our patient has a HLA genotype associated with autoimmune hepatitis.

**Conclusion:**

Patient follow-up will disclose whether our patient suffers from an autoimmune disease and if the presence of anti-soluble liver antigens could precede the development of an autoimmune hepatitis, as the presence of antimitochondrial antibodies can precede primary biliary cirrhosis.

## Introduction

The etiology of autoimmune hepatitis (AIH) is unknown. The detection of non-organ and liver-related autoantibodies in the absence of viral, toxic, metabolic and genetic causes constitutes the hallmark for the diagnosis, but circulating antibodies can be absent in about ten to thirty percent of patients. In routine clinical practice, the International Autoimmune Hepatitis Group scoring system is usually employed. There are two types of AIH. Type 1 AIH (AIH-1) is characterized by the detection of antismooth muscle antibodies (anti-SMA) and/or antinuclear antibodies (ANA). The presence of antineutrophil cytoplasmic autoantibodies (ANCA), antibodies against the asialoglycoprotein receptor (anti-ASGP-R) and anti-soluble liver antigens (anti-SLA) helps in the identification of ANA/SMA-negative patients. Type 2 AIH (AIH-2) is characterized by the presence of autoantibodies against liver kidney microsomalantigens (anti-LKM type 1 or rarely anti-LKM type 3), which contain drug-metabolizing enzymes and/or autoantibodies against liver cytosolic protein type 1 (anti-LC-1). [1].

More than 900 drugs, toxins, and herbs have been reported to cause liver injury, usually with a clinical picture resembling viral hepatitis. It is difficult to identify one drug as being responsible for liver injury because they are often used in combination. At least 24 drugs have been associated with drug-induced chronic hepatitis mimicking AIH (DrAIH). A long interval between drug ingestion and the start of autoimmune signs and symptoms seems to be characteristic. At the time of diagnosis, a histological cirrhotic phase is rarely described. ANA, anti-LKM and SMA have been associated with DrAIH, but they are no longer detectable after drug withdrawal together with biochemical, serological and histologic resolution [2].

Anti-SLA autoantibodies have been demonstrated to be identical to antibodies to liverpancreas antigen (anti-LP) and now they are known as anti-SLA/LP. They are the most specific markers for AIH, particularly in those who lack other autoantibodies. Probably, they were underdetected until standardised immunoassays were available. The anti-SLA target, a ~50 kDa cytosolic enzyme, has been recently identified and efficient commercial ELISA techniques, based on the recombinant antigen, developed. Most authors have found anti-SLA antibodies only in AIH-1 or in cryptogenic hepatitis and never in AIH-2. But others have described anti-SLA positivity in a low percentage of AIH-2 patients. Wies et al recently showed a 30% sensitivity and 100% specificity of anti-SLA for AIH detection [3]. Baeres et al. also described a high specificity if confirmatory Western-blotting and a new recombinant-ELISA were performed [4]. Occasionally anti-SLA antibodies have been found in pediatric AIH-2 and hepatitis C virus (HCV)-infected individuals. These latter findings need confirmatory studies to elucidate the relationship between anti-SLA and HCV infection. Shinoda et al described their presence in patients with high levels of autoantibodies against drug-metabolizing enzymes, which are frequent in DrAIH, but that have been associated with the three groups of liver diseases (AIH-2, DrAIH and viral hepatitis) [5,6].

## Case presentation

A 66-year-old Caucasian woman, with a past history of diabetes mellitus type 2, osteoporosis and no history of liver disease, developed liver dysfunction. She presented with fatigue, progressive jaundice, weight loss of 10 kg and mild epigastric and right upper quadrant abdominal pain over a period of two months. She denied any alcohol or drug abuse or exposure to blood products. She had no antecedents of other autoimmune disorders, and no family history of autoimmune or liver disease. For the previous two years she had received treatment with enalapril but this was stopped as she complained of discomfort. She had also taken metformine for diabetes mellitus, risendronate for osteoporosis, and herbal medicines (Centaurea Aspera L and Coutarea latiflora DC) for hypoglycaemia (self-medicated). Physical examination showed moderate mucocutaneous jaundice without stigmata of chronic liver disease. Blood tests revealed elevated aspartate aminotransferase (AST) 784 U/L (reference interval 0–36), alanine aminotransferase (ALT) 794 U/L (reference interval 0–41), serum bilirubin 6.10 mg/dl (reference interval < 1.40), and _-globulin fraction 26.90% with selective Immunoglobulin G (IgG) elevation (2290 mg/dl). Full blood count, creatinine, urea, electrolytes and pancreatic enzymes were within normal limits. Serological tests for viral hepatitis (HAV, HBV, HCV, CMV, EBV) were negative. alpha-1-antitrypsin phenotype, serum ceruloplasmin, iron and ferritin levels were normal. X-ray tests (abdominal echography and computerized tomography, echoendoscopy) did not show any abnormality. Indirect immunofluorescence (IFI) on HEp-2 cell lines showed ANA with a speckled and cytoplasmic pattern at 1/320 titer. Anti-SMA positivity was evidenced by IFI on rodent liver, kidney and stomach sections ("in house" technique) and corresponded to Factin specificity in an enzyme linked immunoassay (ELISA) from INOVA Diagnostics (San Diego, US). Anti-SLA detection by Dot-blot (D-tek. Mons-BELGIUM) was confirmed by two different techniques; an ELISA with prokaryotically expressed SLA protein (Euroimmun UK. Ltd. Cardiff, UK) and an immunoprecipitation assay (IPA) as described by Costa et al. [7]. HLA (Histocompatibility Leukocyte Antigen) genotyping by PCR-SSP (Dynal Biotech, Oslo, Norway) showed HLA-DRB1* 04; DR B1* 07 genotype.

**Figure 1:**
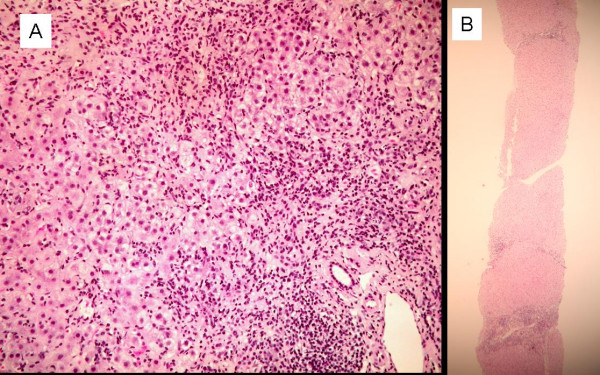
**A**. Initial stage. Severe piecemeal necrosis and lobular damage with severe infiltration of lymphocytes and periportal and pericellular fibrosis. **B. **After drug withdrawal. Chronic hepatitis with mild activity and septal fibrosis.

Tumoral markers (CEA, CA 12.5) were negative except for CA 19.9: 443.2 U/ml (reference interval < 37.0). Liver histology disclosed severe piecemeal necrosis (interface hepatitis), lobular damage manifested by anisonucleosis, ballooning degeneration, hepatocellular swelling and acidophil bodies. Furthermore a severe infiltration of lymphocytes and neutrophils with scarce plasmatic cells and eosinophils, and periportal and pericellular fibrosis, were detected (figure 1A). The inflammatory infiltrates affected most of the portal tracks with fibrosis.

Withdrawal of the medication resulted in normalization of liver function within 3 weeks and according to the AASLD practice guidelines published in 2002, we did not initiate any treatment. Six months later, there was no biochemical or serological alteration, IgG levels were normal and SMA and anti-F-actin were negative. ANA showed only a speckled pattern and anti-SLA remained positive. The second liver biopsy showed chronic hepatitis with mild activity and septal fibrosis, despite clinical and biochemical resolution. Lobular liver cell damage ameliorated, no bridging necrosis or multiacinar necrosis was found and small regenerative hepatocytes were seen (figure 1B).

## Conclusion

On admission, our first hypothesis was that the patient had suffered a drug-induced hepatitis. We speculated that a drug, probably enalapril, was the cause. Nevertheless there are also six cases in the literature of metformin induced hepatitis and some reports of herbal and alendronate-related toxic hepatitis [8]. Our patient did not receive any treatment but we withdrew medication. After that, biochemical parameters fell to reference levels and symptoms disappeared, however anti-SLA autoantibodies remained positive.

The presence of fibrosis, at the time of diagnosis and after 6 months without medication, suggests that the patient was suffering from a silent AIH. It is less probable, as it has been never described, that this was a DrAIH associated with anti-SLA, although DrAIH occasionally can develop fibrosis. Patient follow-up after one year has not revealed any alteration of liver enzymes.

Susceptibility for AIH-1 has been associated with the major histocompatibility alleles DRB1*0301 and DRB1*0401. AIH-2 has different HLA risk factors, the DRB1*07 allele and the HLA haplotype DRB1*15-DQB1*06. Our patient presents two AIH associated alleles: DRB1*04 and DRB1*07. Nowadays, there are not enough data to establish an association between HLA antigens and susceptibility to DrAIH, but perhaps they play a similar role than in AIH because the target antigens (drug-metabolizing enzymes) are common [9].

Our patient showed a moderate elevation of CA 19.9, a tumoral marker that has also been associated with collagen diseases, diabetes mellitus, alcoholic or non-alcoholic liver disease, and acute or chronic pancreatitis. Furthermore, the combined elevation of CA 19.9 and CA 125 is useful for identifying patients with advanced fibrosis or cirrhosis with high specificity.

We concluded that our patient probably presented with a drug-induced hepatitis together with an unknown AIH. Clinical and serological follow-up will allow us to confirm if SLA autoantibodies could, in some cases, be a pre-clinical marker that precedes an autoimmune disease, as the presence of antimitochondrial antibodies can precede primary biliary cirrhosis [10].

## Competing interests

The author(s) declare that they have no competing interests.

## Authors' contributions

AE was the clinical immunologist who wrote the manuscript. AE, AB and CD were the attending physicians responsible for providing all the clinical information. MRJ was the immunologist responsible for the autoimmunity section that performed immunological evaluation and also helped to draft the manuscript. MMC was the pathologist who carried out the histological studies. All authors read, revised and approved the final manuscript.

## Consent

Written informed consent was obtained from the patient for publication of this case report and any accompanying images. A copy of the written consent is available for review by the Editor-in-Chief of this journal.

## References

[B1] Edward L Krawitt MD Autoimmune hepatitis N Engl J Med 2006 354 54 66 10.1056/NEJMra050408 16394302

[B2] Oshomoto K Yamamoto S Drug-induced liver injury associated with antinuclear antibodies Scan J Gastroenterol 2002 11 1345 1346 10.1080/003655202761020687 12465739

[B3] Wies I Brunner S Henninger J Herkel J Kanzler S Mayer zum Büschenfelde KH Lohse AW Identification for SLA/LP autoantibodies in autoimmune hepatitis Lancet 2000 355 1510 1515 10.1016/S0140-6736(00)02166-8 10801173

[B4] Baeres M Herkel J Czaja AJ Wies I Kanzler S Cancado EL Porta G Nishioka M Simon T Daehnrich C Schlumberger W Galle PR Lohse AW Establishment of standardised SLA/LP immunoassays: specificity for autoimmune hepatitis, worldwide occurrences and clinical characteristics Gut 2002 51 259 264 10.1136/gut.51.2.259 12117891 PMC1773315

[B5] Shinoda M Tanaka Y Kuno T Matsufuji T Matsufuji S Murakami Y Mizutani T High levels of autoantibodies against drug-metabolizing enzymes in SLA/LP-positive AIH-1 sera Autoimmunity 2004 37 473 480 10.1080/08916930400001891 15621574

[B6] Manns M Obermayer-Straub P Cytochromes P450 and uridine Triphospatheglucuronosyltrasnferases: Model autoantigens to study Drug-induced, Virus induced, and autoimmune liver disease Hepatology 1997 26 1054 1066 10.1002/hep.510260438 9328334

[B7] Costa M Rodriguez-Sanchez JL Czaja AJ Gelpi C Isolation and characterization of cDNA encoding the antigenic protein of the human tRNA(ser)sec complex recognized by autoantibodies from patients with type- 1 autoimmune hepatitis Clin Exp Immunol 2000 121 364 374 10.1046/j.1365-2249.2000.01280.x 10931155 PMC1905695

[B8] Farrell GC Drug-induced cirrhotic hepatitis Drug-induced liver disease 1994 Edinburgh: Churchill Livingstone 413 30

[B9] Czaja AJ Strettell MD Thomson LJ Santrach PJ Moore SB Donaldson PT Williams R Associations between alleles of the major histocompatibility complex and type 1 autoimmune hepatitis Hepatology 1997 25 317 323 10.1002/hep.510250211 9021941

[B10] Jones DE Autoantigens in primary biliary cirrhosis J Clin Pathol 2000 53 813 821 10.1136/jcp.53.11.813 11127262 PMC1731114

